# Glutamate-reduced diet and ketogenic diet for pediatric drug-resistant epilepsy: a novel approach to treatment

**DOI:** 10.1097/MS9.0000000000001972

**Published:** 2024-03-18

**Authors:** Zaib Un Nisa Mughal, Abdul Malik, Burhanuddin Sohail Rangwala, Hussain Sohail Rangwala, Hareer Fatima, Mirha Ali, Asma A. Farah

**Affiliations:** a Department of Medicine, Jinnah Sindh Medical University, Karachi, Sindh, Pakistan; b Department of Medicine, East Africa University, Bosaso, Somalia

## Introduction

Drug-resistant epilepsy (DRE), also known as refractory or intractable epilepsy, is a condition in which seizures persist despite treatment with appropriate anti-epileptic medications. The incidence of epilepsy is highest in the first 2 years of life. Young children with epilepsy remain at the highest risk of continuing seizures and neurodevelopmental compromise in the long term^1^. Most patients with epilepsy are primarily treated with anti-seizure drugs (ASDs). Thirty percent of patients with epilepsy experience inefficacy of ASDs. In such cases, non-pharmacological treatments, such as ketogenic diet therapy (KDT) and a glutamate-reduced diet, are considered. A ketogenic diet (KD), which is a high-fat low-carbohydrate diet designed to mimic the effects of starvation, is a non-pharmacological treatment option for individuals with DRE^2^. Patients with DRE may undergo dietary therapy if conventional medications fail. Despite successful seizure control and additional benefits, alternative treatments such as nutritional therapy face challenges such as low compliance and palatability. This limited exploration of non-medication options may be due to concerns regarding negative effects and administrative challenges. Acknowledging these aspects can lead to a holistic understanding of epilepsy management. One variant used primarily in adolescents and adults with epilepsy, the modified Atkins diet (MAD), restricts net carbohydrates (subtracting grams of fiber from the total carbohydrate count) to 20 g per day, has high fat and unrestricted protein intake, and has been shown to effectively reduce seizures. Other variants include the classic long-chain triglyceride KD, medium chain, and low glycemic index^3^. A beneficial diet alternative for epilepsy should aim to minimize treatment challenges and adverse effects, while enhancing micronutrient and antioxidant intake.

In the mammalian brain, glutamate plays a pivotal role as the primary neurotransmitter responsible for inducing neural excitation. The proposed mechanism underlying the development of epilepsy involves an imbalance between neural excitement and inhibition. This imbalance renders the brain excessively hyperexcitable, which leads to a phenomenon known as excitotoxicity. Excitotoxicity involves the overactivation of neural cells, causing a decline in their function and resulting in cell death over time. Disruption of the delicate balance between excitation and inhibition contributes to the development and progression of epilepsy^4^. Excitotoxicity, a process in which excessive neural excitation occurs, leads to downstream effects such as oxidative stress and neuroinflammation. In turn, these two phenomena are associated with the development of epilepsy. The interconnected relationship between excitotoxicity, oxidative stress, and neuroinflammation forms what is referred to as neurotoxic trifecta. This concept suggests that these factors mutually reinforce each other, contributing to the complex pathogenesis of epilepsy^5^.

Glutamate, the predominant excitatory neurotransmitter in the central nervous system (CNS), facilitates communication between nerve cells. In typical glutamate transmission, three key steps occur. First, vesicular glutamate transporters (vGLUT1–3) release glutamate and prepare it for exocytosis. The second step involves receptor signaling through glutamate receptors. Lastly, plasma membrane excitatory amino acid transporters (EAAT1–5), primarily located in glial cell processes, facilitate glutamate uptake and clearance from the synaptic cleft. Under normal conditions, glutamate regulates emotional and cognitive functions, and its excessive accumulation activates receptors, leading to structural and functional effects known as glutamate excitotoxicity. These effects include axonal degeneration, dendritic remodeling, and synapse elimination^6^.

KDT is the first-line treatment for pyruvate dehydrogenase insufficiency and glucose transporter 1 deficiency syndrome (Glut1DS). KDTs should be given to children who do not respond well to two anti-epileptic medicines (AEDs) for a variety of epilepsy syndromes, including Dravet syndrome, West syndrome (WS), and epilepsy with myoclonic atonic seizures^7^. KDTs have a wider suitable range than medications due to ketone bodies’ direct inhibition of vesicular glutamate transport, alteration of metabolism through inhibition of increased mitochondrial adenosine triphosphate (ATP) synthesis and glycolysis, ATP-sensitive potassium channel activation to reduce neuronal excitability, and elevation of polyunsaturated fatty acids and reduction of reactive oxygen species through stimulation of mitochondrial dissociation^8^. Thus, in addition to their capacity to decrease neuronal hyperexcitability, KDTs also have neuroprotective actions that address the failure of cellular energy and prevent brain damage from epilepsy^9^.

The efficacy of a reduced-glutamate diet has been demonstrated to alleviate pain, fatigue, cognitive impairment, and mood dysregulation, as shown in previous investigations^8^, which enhances nutrient absorption while minimizing the intake of excitotoxins, especially free glutamate and aspartate. These substances are commonly found in food additives and certain naturally glutamate-rich foods, such as soy sauce, fish sauces, seaweed, tomatoes, mushrooms, and aged cheeses. The reduced-glutamate diet emphasizes the consumption of specific micronutrients and antioxidants known to counteract and mitigate excitotoxicity and subsequent oxidative stress^10^.

The diversity of the gut microbiota significantly affects overall health. In DRE patients, their gut microbiota differs from that of healthy individuals, suggesting a role of dysbiosis in the condition’s development^11^. Dysbiosis may increase susceptibility to seizures and expedite illnesses owing to long-term stress. Restoring a healthy gut microbial community could potentially reduce seizures and improve the quality of life. Additionally, a study found that non-responders to KDT had higher levels of specific bacteria, suggesting that these could be indicators of treatment efficacy and potential therapeutic targets for epilepsy^12^.

A recent meta-analysis examined the efficacy and safety of a KD in treating DRE in pediatric patients. Results from 11 randomized controlled trials (RCTs) involving 788 participants showed significant reductions in seizure frequency and increased odds of achieving seizure freedom with KD interventions, including modified versions such as the MAD and the low glycemic index diet (LGID). The common adverse effects include constipation and vomiting. While KD presents a promising non-pharmacological option for improving the quality of life in non-responsive and non-surgical DRE patients, further research is needed to address its limitations and long-term effects^13^.

Comparing the meta-analysis of ketogenic dietary interventions for pediatric epilepsy with a glutamate-reduced diet, there are notable differences in focus and outcomes. The meta-analysis primarily evaluated the efficacy and adverse effects of KDs (KD, MAD, and LGID), specifically for epilepsy treatment, highlighting positive outcomes such as seizure reduction and freedom. In contrast, a glutamate-reduced diet targets a broader range of health conditions by reducing glutamate intake with the aim of alleviating symptoms associated with conditions such as migraine, fibromyalgia, and mood disorders. Although both dietary approaches aim to improve health, they operate through different mechanisms and have distinct target conditions. The KD induces ketosis and affects neurological conditions such as epilepsy, while the glutamate-reduced diet focuses on minimizing glutamate intake to potentially alleviate symptoms of various conditions. However, a glutamate-reduced diet has shown promising benefits for DRE, including a potential reduction in seizure frequency and severity, as glutamate is implicated in excitotoxicity and neuronal hyperexcitability, which are factors in epilepsy. Further research is needed to fully elucidate the efficacy and mechanisms of action of glutamate-reduced diet, specifically for DRE. Comparing their effectiveness and adverse effects requires separate analyses tailored to each specific dietary approach and target conditions^13,14^.

The meta-analysis revealed the unfavorable consequences associated with employing KDs for pediatric epilepsy. Among the many reported issues, constipation was the most prevalent problem, affecting nearly 39.07% of the children on the diet, which aligns with previous RCTs. In another study, patients on a ketogenic liquid formula were found to have a significantly higher body mass index (BMI) than those taking anti-epileptic medication after 6 months. Other side effects included decreased appetite (18%), lethargy (6%), and nausea (10%). In rare cases, two children experienced recurrent chest infections, while another developed hyperammonemia encephalopathy, both of which resolved when the diet was discontinued. Despite these negative effects, an RCT conducted in 2023 demonstrated that the KD is as effective and well-tolerated as an alternative anti-seizure medication, implying its safety for infants with DRE. For infants who do not respond to two different anti-seizure medications, a KD may be considered as a potential therapeutic option^13,14^.

## Conclusion

The combination of glutamate-reduced and ketogenic diets has shown significant promise in reducing seizure frequency and improving control, surpassing the efficacy of either diet alone. This synergistic effect is attributed to complementary mechanisms that target different aspects of the epileptic network. The glutamate-reduced diet reduces seizure triggers by lowering excitatory neurotransmitter levels, while the KD induces ketosis, providing an alternative fuel source to stabilize neuronal activity. This novel approach offers hope for children with DRE and their families, providing a safe and non-pharmacological alternative to alleviate chronic seizures and enhance their overall well-being.

## Ethical approval

Ethics approval was not required for this editorial.

## Consent

Informed consent was not required for this editorial.

## Sources of funding

The authors received no extramural funding for the study.

## Author contribution

The conceptualization was done by B.S.R. and Z.U.N.M. The literature and drafting of the manuscript were conducted by Z.U.N.M., A.M., H.S.R., H.F., M.A. and A.A.F. The editing and supervision were performed by B.S.R. All authors have read and agreed to the final version of the manuscript.

## Conflicts of interest disclosure

The authors declare no potential conflicts of interest concerning the research, authorship, and/or publication of this article.

## Research registration unique identifying number (UIN)

Not applicable.

## Guarantor

Not applicable.

## Data availability statement

Not applicable.

## Provenance and peer review

Not applicable.
